# The association between dietary polyphenol intake and attention-deficit hyperactivity disorder: a case-control study

**DOI:** 10.1186/s12887-022-03768-3

**Published:** 2022-12-06

**Authors:** Melika Darzi, Khadijeh Abbasi, Reza Ghiasvand, Mohsen Akhavan Tabib, Mohammad Hossein Rouhani

**Affiliations:** 1 Department of Nutrition, Science and Research Branch, Islamic Azad University, Tehran, Iran; 2 Nutrition and Food Security Research Center, Department of Community Nutrition, School of Nutrition and Food Science, Isfahan University of Medical Sciences, Isfahan, Iran; 3 Learning Center, West LA College, Los Angeles, USA

**Keywords:** Polyphenol, Attention-deficit hyperactivity disorder, Nutrition

## Abstract

**Background:**

Previous research found that diets high in fruits and vegetables improved symptoms of attention deficit hyperactivity disorder (ADHD). Nevertheless, the relationship between dietary polyphenol intake and the risk of ADHD was not assessed.

**Objective:**

The purpose of this study was to see if there was a relationship between dietary polyphenol intake and the risk of ADHD in children in preschool and elementary school.

**Methods:**

A total of 400 children aged 4 to 12 years old participated in this case-control research (200 children with diagnosed ADHD and 200 healthy controls). The presence of ADHD was diagnosed according to the Diagnostic and Statistical Manual of Mental Disorders-V criteria. To calculate dietary polyphenol intake, a 168-item food frequency questionnaire and the Phenol-Explorer database were used.

**Results:**

A significant negative association was observed between one unit increase in dietary polyphenol intake and risk of ADHD (OR: 0.995, 95% CI = 0.994 to 0.996, *P* < 0.001) in the crude model. This finding was still significant even after adjusting for body mass index, energy intake, socioeconomic status, gender, and age (OR: 0.992, 95% CI = 0.989 to 0.995, *P* < 0.001).

**Conclusion:**

We found that the increased dietary intake of polyphenols is associated with a lower risk of ADHD in preschool and school children. Prospective studies are needed to corroborate these observations.

## Introduction

Attention-deficit hyperactivity disorder (ADHD) is a common childhood psychiatric disorder characterized by inattention, impulsivity, and hyperactivity [1]. It has been reported that ADHD affects 5% of children worldwide [2]. In Iran, the total prevalence of ADHD in preschool children ranged between 11% and 25.8% [3]. Boys are more likely to be diagnosed with ADHD than girls [4]. ADHD has a variety of negative health consequences and financial hardship for families and communities. Children’s academic achievement and interpersonal relationships can be seriously affected by this disorder [5].

The exact cause of ADHD is unclear. However, genetic and environmental factors have a role in its etiology [6]. Dietary factors have also potential roles in ADHD [7]. A western-style diet, high in fat and refined sugars, has been reported to increase the risk of ADHD. In contrast, healthy dietary patterns like the Mediterranean diet, rich in vegetables, fruits, legumes, whole grains, seafood, and olive oil have been negatively associated with the risk of ADHD [8]. The vegetarian dietary pattern had beneficial effects on ADHD symptoms [9].

Polyphenols are secondary plant metabolites that are grouped into five primary classes based on their chemical structure: phenolic acids, flavonoids, lignans, stilbenes, and others [10]. Polyphenolic compounds have a variety of health benefits, including antioxidant and anti-inflammatory effects [11]. Oxidative stress has been linked to the pathophysiology of psychiatric diseases like depression, and attention deficit hyperactivity disorder (ADHD) [12]. Evidence has suggested that dietary polyphenol intake is associated with mental health and positively affects the brain through its antioxidant properties [13]. Several studies showed that increasing dietary polyphenol intake improved symptoms of ADHD children and had a positive impact on school-aged children’s learning, attention, and behavior [9, 14]. To the best of our knowledge, no investigation has yet been done to show the relationship between dietary polyphenol consumption and the risk of ADHD. In this study, we wanted to evaluate the association between dietary polyphenol intake and the risk of ADHD in preschool and elementary school children.

## Methods

### Design and participants

A case-control study was conducted to compare 200 ADHD patients with 200 non-ADHD controls in Isfahan, Iran in 2017. All participants were 4 to 12 years old. Both groups were matched by sex and age (maximum 6 months age difference). Children with ADHD were recruited from psychotherapy clinics located in the center, north, south, west, and east of Isfahan using a random multistage cluster sampling. Healthy children were enrolled in the study from Isfahan’s preschool and elementary schools. The Diagnostic and Statistical Manual of Mental Disorders-V criteria were used by a psychiatrist to diagnose children with ADHD [15]. The children with ADHD had been diagnosed within the last 6 months and were excluded if they had additional psychiatric disorders. All patients were under a same treatment protocol. The control group was checked for the absence of ADHD or other obvious psychiatric disorders. They were excluded if they had chronic systemic disorders, specific diet plans, or used any medication. All participants and their parents agreed and signed the written informed consent. Isfahan University of Medical Sciences ethics committee Approved the study protocol (IR.MUI.REC.1395.3529).

### Anthropometric measurements

With a non-stretchable tape, the children’s height (cm) was measured to the nearest 0.1 cm. (Seca, Hamburg, Germany). Bodyweight (kg) was measured minimally clothed without shoes on a calibrated scale accurate to 0.1 kg (Seca, Hamburg, Germany). The BMI was computed by dividing the weight in kilos by the height in meters squared.

### Dietary assessment and estimation of polyphenol intake

Dietary intake was examined using a 168-item semi-quantitative interviewer-administered food frequency questionnaire (FFQ) that included a list of foods usually consumed by Iranian children. Parents were asked to provide information on their children’s food intake listed daily, weekly, monthly, or annual throughout the previous year. The FFQ was validated for the Iranian population [16]. A team of nutritionists administered the FFQ. To estimate the amount of food consumption, food portion size images were used. The reported frequency for each food item was converted to a value in number of servings per day. The amount of food consumed was converted to grams by using household measures. A modified version of NUTRITIONIST IV software for Iranian foods was used to analyze total energy and nutrient intake from FFQ (version 7.0; N-Squared Computing).

The following formula was used to calculate total polyphenol intake:

$$\sum\;\lbrack\mathrm{polyphenol}\;\mathrm{content}\;\mathrm{of}\;\mathrm{food}\;\mathrm{item}\;(\mathrm{mg}/100\;\mathrm{gr})\;\times\;\mathrm{amount}\;\mathrm{of}\;\mathrm{consumed}\;\mathrm{food}\;\mathrm{item}\;(\mathrm{grams}\;\mathrm{per}\;\mathrm{day})/100\rbrack.$$

The sum of polyphenols in all food sources in FFQ was used to calculate total individual polyphenol consumption. The Phenol-Explorer database provided us with information on polyphenol concentration in foods (www.phenol-explorer.eu). All foods that contained no polyphenols, or only traces, were eliminated.

### Covariates

As reported by epidemiological studies, sex (male or female) and age (years) are important factors related to ADHD. Considering these confounding factors, a pair-match design was used to match both cases and controls. A questionnaire was used to categorize participants into three groups (low- middle – high) according to their socioeconomic status (SES). Factors queried include family size, education level and employment of both parents, home status, car ownership, and international travel.

### Statistical analysis

The normal distribution of the variables was evaluated using the Kolmogorov-Smirnov test. The independent-samples t-test was used to evaluate between-group differences in weight, height, BMI, and age. The chi-square test was used to assess between-group differences in gender and SES. The multivariate analysis of covariance was used to determine energy-adjusted nutrient intake in both cases and controls. Logistic regression analyses were used to examine the risk of ADHD per one unit increase in dietary polyphenol intake. One crude and two adjusted models were defined in logistic regression. The first adjusted model (model I) was controlled for energy intake and the second model (model II) was additionally adjusted for socioeconomic status, age, body mass index, and gender. *P*-value < 0.05 was defined statistically significant. Statistical Package for Social Sciences version 18 (SPSS Inc., Chicago, Illinois, USA) was used for all statistical analyses.

## Results

The general characteristics of the subjects are reported in Table 1. Between the two groups, there were no significant differences in gender (*P* = 0.490) or age (*P* = 0.439), whereas weight (*P* < 0.001) height (*P* = 0.003) and BMI (*P* = 0.001) were significantly higher in healthy subjects compared with the ADHD group. Also, the socioeconomic status of the two groups differed significantly (*P* < 0.001).

**Table 1:** General characteristics of children with attention deficit hyperactivity disorder and healthy controls

Variable	ADHD (*n* = 200)	Healthy Controls (*n* = 200)	P
Gender (%)			
Male	79.5	76.4	0.264
Female	20.5	23.6	
Age (year)	7.07 ± 1.71	6.93 ± 1.77	0.439
Weight (kg)	20.50 ± 6.43	23.41 ± 9.45	< 0.001
Height (cm)	120.86 ± 9.77	123.97 ± 11.24	0.003
BMI (kg/m^2^)	13.71 ± 2.49	14.69 ± 3.25	0.001
Socio-economic status (%)			
Low	18.0%	1.5%	< 0.001
Middle	65.5%	44.2%	
High	16.5%	54.3%	

*ADHD *Attention deficit hyperactivity disorder, *BMI *Body mass index

Table 2 shows the energy-adjusted dietary intakes of both groups. Dietary intake of protein, fat, saturated fatty acids (SFA), zinc, vitamin A, vitamin B1, fiber, vitamin C, potassium, magnesium, phosphorus, calcium, and polyphenols were significantly higher in the control group (*P* < 0.001 for all). In contrast, the intake of carbohydrates was not different between the two groups (*P* = 0.221).

**Table 2:** Energy-adjusted dietary intakes in children with attention deficit hyperactivity disorder and healthy controls

Nutrient	ADHD (*n* = 200)	Controls (*n* = 200)	P
Carbohydrate (g)	192.26±-41.0	231.47 ± 37.97	0.221
Protein(g)	37.82 ± 11.96	81.08 ± 21.11	< 0.001
Fat(g)	67.24 ± 13.32	64.79 ± 12.07	< 0.001
SFA(g)	18.87 ± 4.03	17.63 ± 3.52	< 0.001
Zinc(mg)	4.46 ± 1.198	8.27 ± 1.87	< 0.001
Vitamin A(mcg)	698.91 ± 284.75	1160.76 ± 335.39	< 0.001
B1(mg)	0.90 ± 0.17	1.33 ± 0.23	< 0.001
Fiber(g)	7.78 ± 2.66	18.43 ± 4.98	< 0.001
Vitamin C (mg)	78.38 ± 27.92	164.85 ± 57.42	< 0.001
Potassium (mg)	1764.46 ± 448.89	3158.88 ± 706.81	< 0.001
Magnesium (mg)	136.67 ± 35.29	254.14 ± 55.84	< 0.001
Phosphorus (mg)	648.02 ± 185.03	1243.34 ± 262.34	< 0.001
Calcium (mg)	462.02 ± 127.71	842.11 ± 190.54	< 0.001
Polyphenols (mg)	648.21 ± 305.67	1641.37 ± 540.30	< 0.001

*SFA *Saturated fatty acids

The risk of ADHD per one unit (mg/100 gr) increase in dietary polyphenol intake is reported in Table 3. The crude model showed an indirect relationship between dietary polyphenol consumption and the risk of ADHD (OR: 0.995, 95% CI = 0.994 to 0.996, *P* < 0.001). Results remained significant after adjusting for energy intake in model I (OR: 0.995, 95% CI = 0.994 to 0.996, *P* < 0.001) and further adjusting for BMI, socioeconomic status, age, and gender in model II (OR: 0.992, 95% CI = 0.989 to 0.995, *P* < 0.001).

**Table 3:** Risk of ADHD per one unit (mg/100 gr) increase in dietary polyphenol intake

Model	Odds ratio	95% CI	*P*	AIC
Crude	0.995	0.994, 0.996	< 0.001	252
Model 1	0.995	0.994, 0.996	< 0.001	365
Model 2	0.992	0.989, 0.995	< 0.001	272

*AIC *Akaike information criterion

Model 1: adjusted for energy intake

Model 2: adjusted for energy intake, socioeconomic status, age, body mass index, and gender

## Discussion

In this case-control study, it was revealed that one unit increase in polyphenol intake could reduce the risk of ADHD in children. Behavioral problems in ADHD can severely diminish academic performance and impair family living and social functioning [17]. Therefore, the consumption of a polyphenol-rich diet may reduce these unfavorable effects.

Relationships between dietary patterns and ADHD have been investigated in previous studies. Healthy dietary patterns characterized by high consumption of fresh fruits and vegetables, whole grains, legumes, vegetable oils, and dairy products would help prevent ADHD or hyperactivity [18, 19]. Also, a vegetarian dietary pattern rich in all types of vegetables, fruits, beans, and grains was negatively associated with ADHD [20, 21]. Other dietary patterns rich in polyphenols, such as the Mediterranean diet and the Dash diet, were negatively correlated with ADHD and improved ADHD symptoms [22, 23]. A cross-sectional study showed that low adherence to the Mediterranean diet may contribute to the development of ADHD [8].

Polyphenols protect against ADHD through several mechanisms. They can modify membrane fluidity and adrenergic receptors [24]. Dietary polyphenols have an antioxidant effect. Pycnogenol, a polyphenolic compound consisting of procyanidins, taxifolin, catechin, and phenolic acids, protects cultured SH-SY5Y neuroblastoma cells against acrolein-induced oxidative stress damages via regulating oxidative stress and raising glutathione levels [25]. Also, pycnogenol may act as a vasodilator, allowing more blood to circulate to the brain and it can regulate the metabolism of catecholamine and inhibit oxidative stress [26, 27]. A clinical trial reported that a mixture of polyphenols in the form of pycnogenol may improve symptoms of ADHD among children [28].

In Iran and most parts of the world, various methods are used to cook vegetables like steaming, boiling, baking, microwaving, or deep frying before eating. These methods influence total polyphenols and their antioxidant capacity [29]. Polyphenols are very sensitive to heat and cooked vegetables have lower total polyphenol contents and antioxidant activity than uncooked ones [30]. Some factors affect cooking like cooking procedure, temperature, duration, and presence of water or moisture. Short cooking duration and/or low temperatures keep more polyphenolic compounds in potatoes [31]. These compounds might be less likely to be destroyed by wet cooking [32]. Frying significantly decreases the total polyphenol content of tomatoes whereas boiling and baking had a small effect [33]. Steaming had higher total polyphenol content in comparison with boiling and microwaving [34]. They are important factors affecting polyphenol concentration and antioxidant activity of vegetables. We may get more benefits from raw vegetables and wet cooking methods.

This study had some limitations. First, we did not consider factors that affect on polyphenol content of foods such as cooking methods. Second, ADHD subtypes were not specified in this study. It is possible that polyphenols intake has different effect on different types of ADHD. Third, the severity of the disease was not assessed, and we just evaluated association between dietary polyphenol intake and risk of the incidence of ADHD disease. Fourth, it is possible that the association between polyphenol intake and risk of the ADHD was significant because of the large sample size, but they are quite small. However, we examined the risk of ADHD per one-unit increase in dietary polyphenol intake. Therefore, we do not expect to observe a remarkable change in the risk of ADHD. Usually, large changes in the risk of a disease are observed in studies that used tertile, quartile or quintile. Another limitation was using FFQ, a dietary assessment tool based on the memories of participants.

We found that increased dietary polyphenol intake was potentially associated with a reduced risk of ADHD. To confirm a causal relationship between dietary polyphenols and the risk of ADHD, clinical trials and cohort studies should be done.

## Data Availability

The datasets used and/or analysed during the current study are available from the corresponding author on reasonable request.
